# Bury my axe in wounded knee: A case of native knee septic arthritis caused by multidrug resistant *Lomentosporaprolificans*

**DOI:** 10.1016/j.mmcr.2026.100774

**Published:** 2026-02-21

**Authors:** Weston Carpenter, Catherine Pulaski, Alexander Hahn, Brenton Nash, Michael Sobieraj, Jeffrey Aeschlimann, Lisa M. Chirch

**Affiliations:** a University of Connecticut School of Medicine, United States; b UConn Health Department of Medicine & Division of Infectious Diseases, United States; c UConn Health Department of Orthopedic Surgery, United States; d University of Connecticut School of Pharmacy, United States

**Keywords:** Fungal septic arthritis, *Lomentospora prolificans*, Olorofim

## Abstract

A 39-year-old male presenting with swelling and pain in the left knee after traumatic injury was diagnosed with rapidly progressive septic arthritis of the knee due to pan-resistant *Lomentospora prolificans*. While *L. prolificans* is an environmental mold, it infrequently causes infection in immunocompetent patients. These hosts most commonly suffer soft tissue or osteoarticular infections following direct, often traumatic inoculation, as opposed to the disseminated infection seen in patients with profound immunocompromise. This report describes a case of osteoarticular infection following traumatic inoculation in an immunocompetent host. Following several unsuccessful attempts at repeated incision and drainage alongside salvage antifungal therapy, the patient was successfully treated with olorofim, a novel investigational antifungal agent.

## 1 Introduction

Septic arthritis of the native joint has a prevalence of about 1 to 35 cases per 100,000. This number varies based on age, location, immunocompetency, pre-existing joint disease, injection drug use, ethnicity, and socioeconomic status [[1], [2], [3], [4], [5], [6], [7]]. Bacterial septic arthritis composes the vast majority of cases, with spread of the infectious organism to the joint often occurring via hematogenous seeding, direct entry of bacteria into the joint, or spread from an adjacent soft tissue or bone [8,9]. Rarely, however, septic arthritis may result from fungal infection. This should be suspected in a patient who presents with persistent culture-negative oligoarthritis or monoarthritis, especially if symptoms arose shortly after a trauma or penetrating wound.

While the prevalence of fungal arthritis is much lower than that of bacterial etiologies, notable fungal organisms that can cause joint infection include *Sporothrix schenkii*, *Coccidioides* spp, and *Candida* spp. However, many others, including environmental molds such as *Aspergillus*, *Scedosporium*, and *Lomentospora* also have the potential [10] to trigger septic arthritis. These molds are opportunistic pathogens associated with a wide range of pathologies, particularly in severely immunocompromised hosts. Inoculation occurs via two main routes: conidial inhalation or direct conidial entry into tissues. Osteoarticular infection can occur via secondary dissemination from a pulmonary portal of entry (more common in the immunocompromised) or via traumatic inoculation (more common in the immunocompetent) [10]. This paper presents the case of a 39-year-old immunocompetent male who developed osteoarticular infection following traumatic inoculation and subsequent infection by *L. prolificans*, a common environmental mold and recognized human pathogen.

## 2 Case presentation

**Fig. 1:**
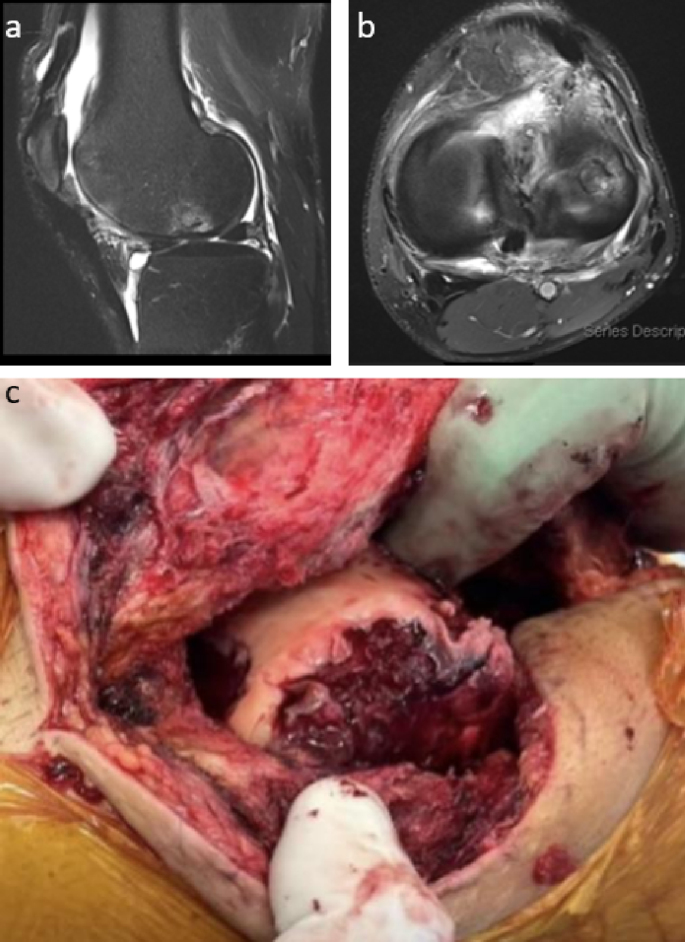
**Gross intraoperative appearance and MRI findings during initial presentation.** Both (a) sagittal and (b) axial MRI imaging of the left knee revealed moderate-sized joint effusion with enhancement and thickening of the synovial lining, as well as foci of cortical irregularity and bone marrow edema suggesting septic arthritis. (c) Initial surgical washout of the knee yielded extensive exudate following debridement of the joint capsule and synovium.

**Fig. 2:**
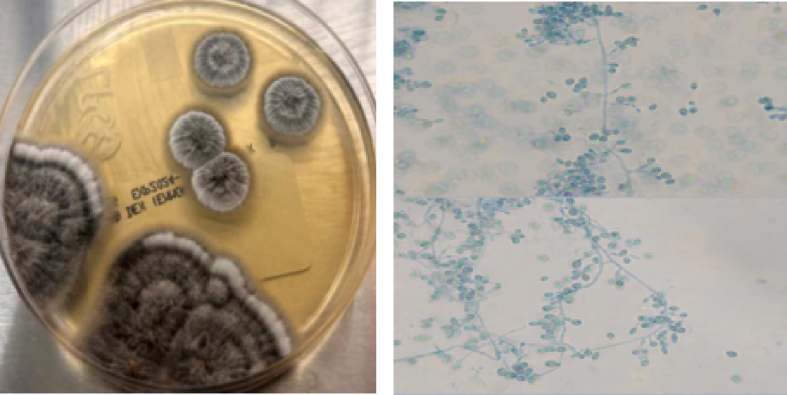
**Left knee arthrocentesis fungal culture growth seven days after collection.** Pale mycelial colonies were evident on Sabouraud dextrose, inhibitory mold, and brain heart infusion agars by day 4 after inoculation. Mature gray-black, wooly colonies were noted by day 7, with colony reverse becoming dark over time. Lactophenol preps revealed abundant large, smooth-walled, oval conidia borne singly on short, tapered conidiogenous cells located individually or in small aggregates. No cleistothecia nor graphium forms were observed. (Images and culture interpretation courtesy of P. Rocco LaSala, MD, UConn Health).

A 39-year-old male presented with edema, erythema, and pain in the left knee one month after repair of a left distal thigh laceration he sustained while cutting wood with an axe in his yard in rural New England. The wound had been closed at the initial encounter in the emergency department (ED) and the sutures were removed eight days following the injury with no signs of infection. However, he presented to urgent care two weeks after the initial injury (day 0) complaining of increasing left knee pain and limited range of motion (ROM). After an examination revealed edema and decreased ROM without warmth or erythema, he was trialed on empiric cephalexin by the outside urgent care facility but returned to the ED one week later (day 7) due to worsening swelling and increasingly limited ROM. Subsequent arthrocentesis showed 30,115 nucleated cells (76% neutrophils), no crystals, and a negative gram stain. MRI of the knee (Fig. 1a–b) indicated joint effusion with synovial enhancement, partial laceration of the quadriceps tendon, and evidence of a prior Osteochondral Autograft Transfer System (OATS) procedure. He was eventually discharged from the ED after a change in his antibiotic regimen to daily levofloxacin but returned nine days later (day 16), with arthrocentesis once again showing high numbers of nucleated cells, increased neutrophil concentrations, and a negative gram-stain. Subsequent synovial fluid culture from the visit on day 7 yielded growth of a mold after seven days incubation (Fig. 2), later identified as *L. prolificans* by Associated Regional and University Pathologists (ARUP) Laboratories via matrix-assisted laser desorption/ionization time of flight (MALDI-TOF) mass spectrometry. The initial growth of mold prompted discontinuation of antibiotics and hospital admission for further evaluation and antifungal treatment.

Following admission to the hospital, the patient was initiated on voriconazole (6 mg/kg IV loading dose, then 200 mg orally twice daily) therapy while awaiting further microbiological information on the isolate. This was later adjusted to include triple antifungal therapy with voriconazole (no change in dosage), terbinafine (250 mg orally twice daily), and caspofungin (70 mg IV loading dose, then 50 mg IV daily), with the goal of a synergistic effect [11]. Nevertheless, this salvage antifungal therapy, alongside serial debridements (Fig. 1c), failed to yield significant improvement in the patient's condition. Multiple cultures obtained with each procedure were positive for mold growth. In total, the patient underwent nine irrigation and drainage (I&D) procedures over the course of two months after the initial injury. The first was performed arthroscopically one month after initial presentation and followed by an open lateral parapatellar approach one week later. This approach angle was chosen due to prior knee surgery making a medial parapatellar approach infeasible. The remaining surgeries were performed open, with the final procedure including placement of an articulating antimicrobial impregnated spacer (1200 mg voriconazole).

Given the lack of clinical improvement after extensive surgical intervention, as well as susceptibility testing that showed pan-resistance to all traditional antifungals (including terbinafine, voriconazole, itraconazole, posaconazole, fluconazole, amphotericin B, micafungin, caspofungin, and anidulafungin), investigation into other treatment options was pursued. An Emergency Investigational New Drug (EIND) application for the novel antifungal olorofim was initiated with both the FDA and the pharmaceutical company F2G Ltd. Expedited IRB approval was obtained, with drug arrival occurring approximately two weeks after initiation of the approval process. Antifungal susceptibility testing with respect to olorofim was also requested [Minimum Inhibitory Concentration (MIC) 0.125 mcg/ml, University of Texas HSC, San Antonio].

The patient underwent his last I&D procedure that admission within days of starting olorofim (150 mg oral loading dose every 12 hours for 1 day, then 90 mg orally every 12 hours), and only one positive culture was obtained out of several collected. He remained on olorofim from discharge in late November 2024 through a subsequent revision with placement of a destination spacer in February 2025. This procedure took place approximately five months from the initial injury, and all cultures collected intraoperatively were negative. Olorofim was well tolerated by the patient; close monitoring of liver function tests (LFT) revealed repeatedly normal results. C-reactive protein, which had been elevated at 210 mg/L, was repeatedly normal (<0.4 mg/L) after the February revision. On outpatient follow-up, the patient has continued to improve in terms of mobility and function and has participated actively with physical therapy. He completed approximately six months of total therapy with olorofim in July 2025 and had no evidence of recurrent infection over the ensuing four months. The patient recently underwent left revision total knee arthroplasty without complication in November 2025, just over one year after the initial injury. In post-operative follow-up he is healing well without signs or symptoms of recurrent infection and is ambulating without assistance.

## 3 Discussion

This report details the case of an immunocompetent patient who developed a monoarticular septic arthritis following traumatic inoculation of the knee by *L. prolificans*. Formerly *Scedosporium prolificans* and/or *Scedosporium inflatum*, *L. prolificans* has the capacity to infect both immunocompetent and immunocompromised hosts. Patients with a robust immune response most often suffer localized soft tissue or osteoarticular infections following direct inoculation by this fungus, usually through trauma. Systemic infection may occur in immunocompromised patients and is often fatal in transplant patients or those suffering from prolonged neutropenia [12]. Treatment of *L. prolificans* is often challenging due to its intrinsic resistance to most commonly available antifungals, as confirmed by susceptibility testing in this case. However, novel agents are under investigation, including the first-in-class dihydroorotate dehydrogenase inhibitor olorofim [13]. Case reports describe treatment of septic arthritis of the knee due to *Scedosporium apiospermum* with olorofim [14], but *L. prolificans* is often more aggressive and has poorer outcomes [15]. This case represents a unique example of a clinically significant fungal joint infection in an unlikely patient that required the use of olorofim after failure of traditional antifungal therapy.

Treatment of septic arthritis is multifaceted. Patients first require drainage of the joint, whether that be needle aspiration, arthroscopy, or arthrotomy in an open surgical setting [16]. Since most cases of septic arthritis are bacterial in nature, initial treatment of the infection involves empiric antimicrobial therapy designed to cover the most likely pathogens based on gram stain, usually anti-staphylococcal and/or additional gram-negative coverage. For the less common fungal arthritis, surgical drainage remains a mainstay in treatment. However, pharmacological management requires the use of combination antifungal therapy. Historically, amphotericin B has been first-line, but development of new azoles (ex. voriconazole) and echinocandins have increased the number of available options, including combination therapy [11]. Nevertheless, the limited bone penetration of many antifungal drugs and steady increase in antifungal resistance has made treating fungal septic arthritis challenging. Furthermore, since the number of patients who are afflicted by osteoarticular fungal infections is low, limited data is available to aid in the development of new medications and/or treatment algorithms.

The management of *L. prolificans* in particular can be challenging due to its significant antifungal resistance. The resistance mechanisms of *L. prolificans* are similar to other fungal species but are enhanced by the creation of a biofilm that can bind antifungal agents and prevent them from reaching their targets. Some antifungal agents, especially the -azoles and polyenes, bind preferentially to the extracellular matrix of the biofilms rather than their targets within fungal cells, thus decreasing their treatment efficacy [17]. Empiric therapy typically consists of voriconazole in combination with surgical debridement of infected tissues, but definitive therapy must be determined by evaluation of resistance testing. Case reports have noted some success with the combination of terbinafine with voriconazole alongside surgical intervention [18,19]. However, in cases in which the organism is resistant to all commonly used agents, such as this one, novel and investigational antifungals must be considered.

Olorofim is the first of the novel class of orotomide antifungals that acts through inhibition of the mitochondrial membrane-associated dihydroorotate dehydrogenase enzyme. This enzyme plays a key role in pyrimidine biosynthesis within a number of clinically relevant fungi [20], making it an attractive target for antifungal therapies. In addition, olorofim has been shown to exhibit significant antibiofilm activity against *L. prolificans*. A recent publication describes the clinical experience with olorofim from an open-label Phase 2b study [20]. Among 26 patients who had invasive *L. prolificans* infections, nearly half (42%) achieved the primary outcome measure (either complete or partial response to olorofim administration) at day 42 of therapy. Fourteen of these cases had infection involving soft tissues, such as the lung or nasal cavity, but the remaining twelve patients were reported with *L. prolificans* involvement of either a bone or joint. Although olorofim was generally well-tolerated during the study, drug-induced liver injury was observed in 22% of the 203 patients, with liver injury being considered “at least possibly related to olorofim” in 10% of the patients, suggesting that monitoring liver enzymes while on olorofim is good clinical practice. Finally, similar to azole antifungals, olorofim inhibits hepatic CYP3A4 enzymes, so careful screening of concomitant medications is required.

In this case, the patient presented with the signs and symptoms of septic arthritis but had repeatedly negative synovial fluid bacterial cultures. Expansion of the differential diagnosis to include fungal septic arthritis proved successful, with subsequent cultures positive for *L. prolificans*. However*, L. prolificans’* inherent resistance to traditional antifungal therapy complicated management of this patient. Continued attempts at treatment with both surgical debridement and combination antifungals was unsuccessful. Long-term treatment with olorofim, a novel orotomide antifungal, was well tolerated, and along with aggressive surgical management resulted in resolution of the osteoarticular infection and successful total knee replacement.

## CRediT authorship contribution statement

**Weston Carpenter:** Writing – review & editing, Writing – original draft. **Catherine Pulaski:** Writing – original draft, Conceptualization. **Alexander Hahn:** Writing – original draft. **Brenton Nash:** Writing – review & editing, Writing – original draft, Conceptualization. **Michael Sobieraj:** Writing – review & editing, Supervision, Conceptualization. **Jeffrey Aeschlimann:** Writing – review & editing, Writing – original draft. **Lisa M. Chirch:** Conceptualization, Resources, Supervision, Project administration, Writing – review & editing.

## Ethical form

Please note that this journal requires full disclosure of all sources of funding and potential conflicts of interest. The journal also requires a declaration that the author(s) have obtained written and signed consent to publish the case report report/case series from the patient(s) or legal guardian(s).

The statements on funding, conflict of interest and consent need to be submitted via our Ethical Form that can be downloaded from the submission site www.ees.elsevier.com/mmcr. **Please note that your manuscript will not be considered for publication until the signed Ethical Form has been received.**

## Consent

Written informed consent was obtained from the patient or legal guardian(s) for publication of this case report and accompanying images. A copy of the written consent is available for review by the Editor-in-Chief of this journal on request.

## Funding source

There are none.

## Conflict of interest

There are none.
